# Preference by Donkeys and Goats among Five Mediterranean Forest Species: Implications for Reducing Fire Hazard

**DOI:** 10.3390/ani10081302

**Published:** 2020-07-30

**Authors:** Jordi Bartolomé, Jordi Miró, Xavier Panadès, Maria José Broncano, Josefina Plaixats, Teresa Rigau, Maria José Milán, Elena Baraza

**Affiliations:** 1 Departament de Ciència Animal i dels Aliments, Grup de Recerca en Remugants, Universitat Autònoma de Barcelona, 08193 Bellaterra, Spain; mj.broncano@creaf.uab.cat (M.J.B.); josefina.plaixats@uab.cat (J.P.); mariajose.milan@uab.cat (M.J.M.); 2 Departament de Medicina i Cirurgia Animal, Universitat Autònoma de Barcelona, 08193 Bellaterra, Spain; teresa.rigau@uab.cat; 3 Museu de la Conca Dellà, C/ del Museu 4, 25650 Isona, Spain; xpan100@gmail.com; 4 Departament de Biologia, Universitat de les Illes Balears, Cra. de Valldemossa km 7.5, 07122 Palma, Spain; elena.baraza@uib.es

**Keywords:** cafeteria test, *Quercus ilex*, *Pinus halepensis*, *Phillyrea latifolia*, *Rubus ulmifolius*, *Brachypodium retusum*

## Abstract

**Simple Summary:**

Donkeys and goats are animals adapted to graze in understories, and thus contribute to the prevention of forest fires. In this work, the preferences of donkeys and goats have been determined for five key plant species of the Mediterranean forest, where large forest fires have increased as a result of global change. Using a multiple selection test, it has been observed that both species can complement each other, since donkeys consume more fine fuel, such as *Brachypodium retusum*, and goats, highly flammable woody species, such as *Pinus halepensis*. In this way, browsing becomes an ecosystem service, which, in the case of donkeys, can even help prevent their extinction.

**Abstract:**

During the second half of the 20th century, European countries experienced an increase in their forest area due to the global change. Consequently, there has been an increase in large forest fires, mainly in the Mediterranean basin, and this has forced the development of several types of prevention programs. One of them is the control of the understory by livestock. In this sense, browsing with a combination of donkeys and goats could be a good option, as both animals usually feed on forest species. However, little is known about their preferences for the key species of the Mediterranean forest. Using a cafeteria test, the preferences and consumption of both animals have been determined for five typical species of the Mediterranean forest, such as *Quercus ilex*, *Pinus halepensis*, *Phillyrea latifolia*, *Rubus ulmifolius,* and *Brachypodium retusum.* Results showed that donkeys and goats could act complementarily in the reduction of the fuel biomass of forests. Donkeys appear to act more on fine fuel, such as *B. retusum*, and goats on the more pyrophyte species, in this case *P. halepensis*. In addition, given that donkeys are at severe risk of extinction in Europe, this role of providing ecosystem services could contribute to their conservation. Despite this study only showing that goats and donkeys would consume all five presented plant species and that there are some differences in consumption during a short-term test, it constitutes a useful first step for conservation and fire prevention in the Mediterranean forests.

## 1. Introduction

There has been massive depopulation in rural areas due to the intense socio-economical changes since the industrial revolution, as well as the reduction of subsistence and traditional agriculture, forestry, and animal husbandry [1]. One of the consequences of this process has been that Europe, North America, and part of Asia experienced an increase in their forest area during the second half of the 20th century [2]. The forests have accumulated into a thick carpet of biomass with highly combustible undergrowth, which ultimately has contributed to the recurrent and virulent forests fires in the Mediterranean basin [3]. In recent decades, fires in Mediterranean Europe have become larger and more frequent [4]. In the past, livestock used to graze the undergrowth, even if it was dedicated to logging, looking for the scarce food resources that the forest produces, and contributing to diminishing the fire hazard.

Grazing programs in forests from the private and public sectors have currently been implemented in some Mediterranean areas, with the explicit intention of reducing fire risk [5,6,7]. These have shown that grazing management of forest vegetation not only prevents fires, but also preserves or increases ecosystem biodiversity, activates the rural economies (e.g., ecotourism, controlled hunting), and enhances scenic qualities [8,9]. In addition, control of the undergrowth by livestock is considered the most cost-effective treatment, even though it requires certain investments (fences, water supplies, forage supplementation), and occasionally needs to be combined with another method [10,11].

Different livestock species have been used as a control tool for the Mediterranean understory. Small ruminants, such as sheep and goats, have often been used due to their hardiness and ability to include a wide variety of woody species in their diet [12]. Due to their more selective nature, and their ability to tolerate many secondary compounds [13], goats are surely the most widely used animal for this purpose (e.g., Mancilla-Leytón et al. [5]; Lovreglio at et al. [6]). For instance, several organizations have recently appeared in California, dedicated to renting their goats to clear public and private land in order to prevent fire risk (e.g., https://www.citygrazing.org/). However, limitations in the cut diameter of bites, the difficulty of penetrating very dense understories, and the avoidance of grasses by goats [6] have led, in many cases, to larger livestock to be preferred for forest management. Bovines and equines can browse in thicker branches than small ruminants, and add the greatest effect of trampling on vegetation. However, the cattle effect is limited by their preference for herbaceous plants [14] and by the quality of the food offered by the Mediterranean understory [15]. The equines are well suited to reduce grass encroachment as they can graze in low-quality fodder [16,17]. It has been observed that horses and goats grazing together in a Mediterranean scrub reduced plant phytovolume significantly [18]. Lamoot et al. [19] showed that donkeys preferred a grassy habitat for foraging in all seasons. Donkeys have also been used successfully to control flammable grasses in the Mediterranean scrub [20]. Along these lines, the combination of donkeys and goats seems to be a good option to control the biomass of the Mediterranean understory. However, donkeys were the livestock most affected by the industrial revolution, and with the upheaval in agriculture, most European donkey breeds are disappearing [21]. In this sense, providing the species with an ecosystem service role could strengthen its survival. Finally, the success of this type of management will depend on the preferences of both animals for the most abundant and most flammable species in the forest. There is currently very little information on this aspect. For this reason, the objective of this work was to determine preferences, and compare the consumption of donkeys and goats for key plant species of the Western Mediterranean forests.

## 2. Materials and Methods

### 2.1. Plant Species

Five plants were offered to animals during the experiment: *Quercus ilex*, *Pinus halepensis*, *Phillyrea latifolia*, *Rubus ulmifolius,* and *Brachypodium retusum*. These are important components of the Western Mediterranean forests. They are all evergreen, so their twigs and leaves are available year-round. *Q. ilex* and *P. halepensis* are the most common tree species in these forests. Both are highly flammable [5,22]. In addition, *P. halepensis* is a pyrophyte species, with several traits that facilitate its dispersion after fire [23]. *Ph. latifolia* and *R. ulmifolius* are common shrubs in the understory. The first is a deep-rooted shrub distributed in the warmer and drier Mediterranean basin, and with good resilience after fires [24]. The second can become dominant after fire, especially if it is not affected by grazing [25]. Both facilitate the spread of fire from the understory to the canopy of trees. *B. retusum* is a grass usually abundant in the herbaceous layer, and constitutes fine fuel for the initiation of many fires [26].

### 2.2. Animals and Feeding Trials

The study was performed with seven Catalan donkeys (*Equus asinus*) and seven female Murciano–Granadina goats (*Capra hircus*) at the Experimental Farm of Autonomous University of Barcelona (Spain). Both are autochthonous breeds of the Iberian Peninsula. Animals were 3–8 years of age in the case of donkeys and 1–2 years in the case of goats, and their initial body weight was 375 ± 25 and 39 ± 6 kg for donkeys and goats, respectively. These animals were selected in a pre-experiment from two herds of 15 donkeys and 30 goats, obtaining the average values of preference on basic feeds, such as barley (*Hordeum vulgare*), alfalfa (*Medicago sativa*), and rye-grass (*Lolium multiflorum*), offered in the same proportion, of the whole herd. The seven animals that presented the values closest to the mean were chosen. All of them were in excellent condition and remained at a constant live weight throughout the experiment. No more animals were used in the experiment for logistical reasons. In order to avoid biases from acquired diet preferences [27], the selected animals had not previously grazed in a Mediterranean forest. In addition, they were given a 5-day adaptation period to adjust to the 3 × 4 m individual boxes environment. During this period, each morning the animals remained in the boxes for 15 min without any food supply.

Every day, animals were placed in the individual boxes, and were tested separately, and in turn. Each one could consume 500 g of fresh forage of each plant species for 15 min undisturbed. It was considered that 15 min was enough time for the animals to finish the offered food. The plant material consisted of bundles of branches less than 5mm in diameter, collected daily from the forest near the farm, and tied to a metal bar located within reach of the animals’ mouths. Plant material in the box was randomly changed every day to avoid bias by side preferences of certain animals. Species availability allowed animals to make their own choices. The first choice was recorded as an estimation of preferred species [28,29]. An observer (the same person in all trials) standing in the neighboring enclosure recorded the choices made by each animal. Consumption was calculated by subtracting the remaining fresh weight from the initial weight. These cafeteria trials were conducted between 0800 and 1100 in the morning. The order in which animals were tested varied randomly every day to prevent conditioned patterns. The animals were within sight of companions during tests to minimize isolation and stress. The experiment was carried out for a period of fifteen consecutive days. After the feeding trial, animals were reincorporated to the corresponding stable, where the rest of the farm herd was. Once there, the herd received a basic diet of concentrated feed and barley straw in its feeder. All animals had free access to water and salt blocks. According to McArthur et al. [27], it is important for animals to receive a basic diet in order to test preferences between plant species under conditions in which nutritional or energetic requirements are satisfied.

### 2.3. Legal Requirements

The experiment complied with the Decree 214/97 of the Catalan Autonomous Government regarding the use of animals in scientific experiments. It was authorized by the Institutional Animal Care and Use Committee of the Autonomous University of Barcelona.

### 2.4. Statistical Analysis

Multivariate analysis of variance (MANOVA) with repeated measurements was used to determine whether there were any differences in the fresh weight consumed (dependent variable) among plant species and between animals throughout the 15 days of experimentation. Time (days) was the repeated factor, and the time × species interaction term was used to recognize differences among plant species in consumption patterns over time.

The analysis of the first plant species chosen every day by the animals was carried out using a logistic regression model for nominal responses. JMP7 software (Version 7.0: SAS Institute, Inc., Cary, NC, USA) was employed in this statistical analysis.

## 3. Results

**Figure 1:**
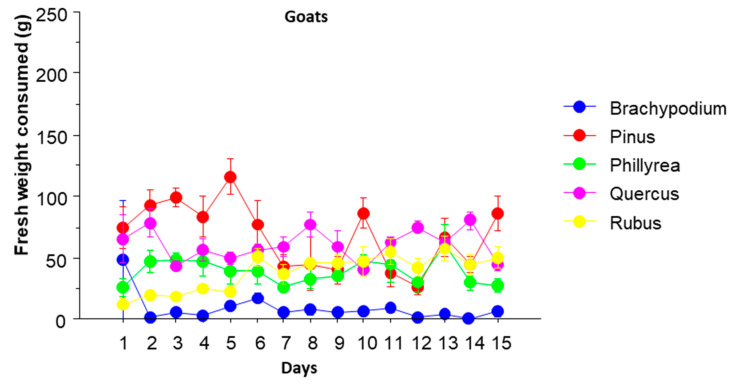
Daily average of fresh weight consumed by goats in the choice tests (*n =* 7). Lines crossing the circles are standard errors.

**Figure 2:**
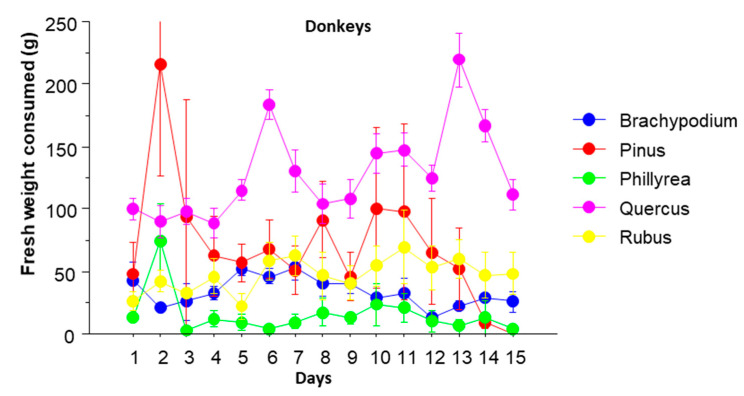
Daily average of fresh weight consumed by donkeys in the choice tests (*n* = 7). Lines crossing the circles are standard errors.

There were no significant differences in the amounts of plant material consumed between both animal types (MANOVA, *p* = 0.1915), despite a clear difference in size between both species. This can probably be explained by the difference in the consumption rate between both species. Thus, Hoffmann et al. [30] and Lamoot et al. [19] got ranges of 8 to 15 and 9 to 12 bites per minute respectively for donkeys, and Negui et al. [31] and Rodrigues et al. [32] got ranges of 15 to 31 and 19 to 24 bites per minute, respectively, for goats. Figure 1 and Figure 2 show the evolution of the consumption of each species over time by goats and donkeys, respectively.

Significant differences were obtained in the consumption of the different species, with *Q. ilex* being the most consumed, and *B. retusum* the least (MANOVA, *p* < 0.0001). Significant differences were also obtained for the interactions between animal type and plant species (MANOVA, *p* < 0.0001), animal type and time (MANOVA, *p* = 0.0004), time and plant species (MANOVA, *p* < 0.0001), and for the interactions of the three factors (MANOVA, *p* < 0.0001).

The species that was consumed the most by donkeys were *Q. ilex,* and by goats, *P. halepensis* and *Q. ilex*. The species least consumed by donkeys was *Ph. latifolia,* and by goats it was *B. retusum*. Donkeys consumed more *B. retusum* and *Q. ilex* than goats, and goats more *Ph. latifolia* than donkeys. The amount of plant consumed throughout the time is more constant in goats than in donkeys. The only plant species in which the amount consumed did not vary significantly throughout the 15 days was *Ph. latifolia*. The consumption of *Q. ilex* and *R. ulmifolius* increased during the firsts five days and then remained high, with a peak towards the last days. The amount of *B. retusum* oscillated, with a maximum consumed the first day, and a minimum consumed the second, then increased again until the sixth day and from that decreased again. Finally, the consumption of *P. halepensis* was decreasing with small oscillations throughout the period.

Goats consumed more *Ph. latifolia* than donkeys for almost the whole period, and more *P. halepensis* mainly during the first half of the period. On the other hand, donkeys ate much more *B. retusum* than goats, with the only exception of the first day. They also ate more *Q. ilex* during the whole period.

**Figure 3:**
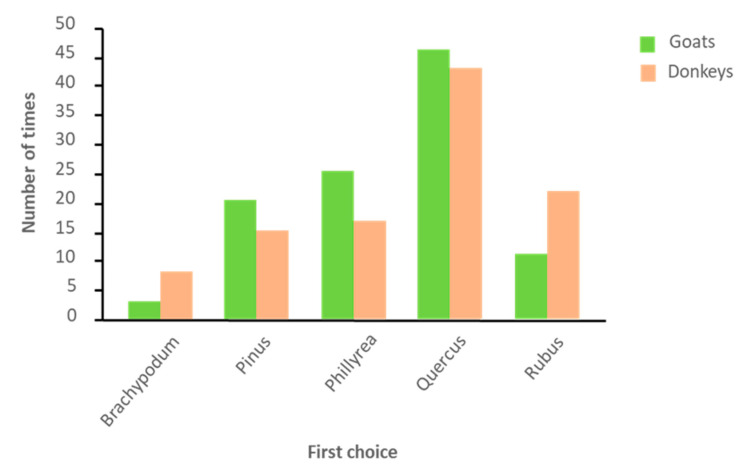
Total number of times each plant species was chosen as the first option.

There was no significant difference observed in the first choice of the animals. Clearly, *Q. ilex* was the first chosen in most times, and *B. retusum* was the last (Figure 3). Despite this, the first choice of goats was more constant between animals and between days than in donkeys. A couple of donkeys never chose *P. halepensis* as a first choice, another never chose *B. retusum,* and another one never chose *Ph. latifolia*. On the other hand, there was a donkey that chose this last species for most of the days. Donkeys chose *Ph. latifolia* as the first option, mainly during the first days, *Q. ilex* mainly during the second half of the period, and *B. retusum* mainly during the intermediate days.

## 4. Discussion

This study is an encouraging first step in a much longer process to establish if goats and donkeys could really reduce fire hazards in Mediterranean forests. From the results obtained, it can be concluded that donkeys and goats could act complimentarily in the reduction of the fuel biomass of forests. Both species have a long history of browsing on Mediterranean shrub ranges, and both animals consumed all the plant species considered, although in different ways. The higher consumption and higher preference for *Q. ilex*, as well as the lower consumption and lower preference for *B. retusum* could be related to their nutritional value. The dominant woody species in the Mediterranean basin are generally of low nutritional quality and contain secondary metabolites, such as tannins, terpenes, and volatile oils [33,34]. Nonetheless, these woody species are often selected by grazing animals, because their leaves have more protein and less fiber than leaves and stems of grasses [35,36]. *Q. ilex* is known to have a higher crude protein content and lower fiber content than *B. retusum* [34]. *R. ulmifolius* also has an acceptable crude protein content [37], and this could possibly explain the consumption increase over the days in both animals, as happened with *Q. ilex*. The higher consumption of *Q. ilex* by donkeys could perhaps be explained by the differences in the bite size of both animals. However, both species have physical defenses, small spines on the leaves of *Q. ilex* and spikes on the stems of *R. ulmifolius*. Several studies have reported on the influence of spinescence in shrubs on the feeding behavior of mammalian browsers [38,39]. Apparently, in this case, these defenses do not represent an impediment for either animal.

Goats consumed *P. halepensis* at the same level as *Q. ilex*, despite the content of secondary compounds that this species contains. *P. halepensis* is a terpene-storing plant [40]. Something similar occurs with *Ph. latifolia*, with a high number of biologically active substances, such as glycosides and terpenes [41], and it was consumed more by goats than by donkeys. This is surely due to the salivary secretions of goats containing proline-rich proteins that bind some of these secondary compounds, thus alleviating their aversive effects [13].

On the other hand, the fact that donkeys consumed more *B. retusum* could be related to their ability to eat foods with high fiber content and low nutritional quality. Equids can compensate for low-quality fodder through increased fodder uptake [42]. The ability of donkeys to reduce biomass of easily flammable grasses has been highlighted by Gulias et al. [20], in the case of the tall grass, *Ampelodesmos mauritanica*. Control of dominant tall grasses by other non-ruminants, such as horses, was also shown in sub-Mediterranean mountain pastures in Italy [43]. For its part, the preference in goats seems to be more affected by the fiber content than by secondary compounds [44].

Another interesting result is the fact that goats, compared to donkeys, had more constant consumption and preferences over time, and with less variability between individuals. This is surprising because goats tended to select a mixed diet when different food species were simultaneously available, and this has been suggested as a foraging strategy for reducing the risk of toxicity [45].

Finally, some limitations of this study, related to the fact that the study was carried out under controlled conditions and not in the field, should be explicitly mentioned. This includes the fact that there may have been novelty effects of using animals who had never consumed those plant species. The relative attractiveness may vary when animals have more options, such offered by an actual forest situation. In addition, animals were tested individually, looking for possible differences between individuals, but it should be recognized that social effects could alter feed consumption in highly gregarious animals like goats and donkeys in field conditions.

## 5. Conclusions

In summary, considering the consumption and preferences of both animals, the use of mixed herds of donkeys and goats as a tool for controlling biomass fuel allowed us to predict an effect on key species in fires in the Mediterranean forest. Donkeys acted on fine fuel, such as *B. retusum*, while goats acted on the more pyrophyte species, such as *P. halepensis*. However, caution is required in extrapolating these results to free-ranging situations. Obviously, much larger studies (more animals, exposed to the feed for much longer each day and considering Mediterranean seasonality) are essential to provide a more realistic idea of how actual implementation would work.

Finally, it is worth noting that since the donkey can be a good manager of the Mediterranean forest, this is another possible strategy for the conservation of endangered breeds.
